# The CCHamide 1 receptor modulates sensory perception and olfactory behavior in starved *Drosophila*

**DOI:** 10.1038/srep02765

**Published:** 2013-09-26

**Authors:** Abu Farhan, Jyotasana Gulati, Ewald Groβe-Wilde, Heiko Vogel, Bill S. Hansson, Markus Knaden

**Affiliations:** 1 Department of Evolutionary Neuroethology, Max Planck Institute for Chemical Ecology, Hans-Knöll-Str. 8, 07749 Jena, Germany; 2 Department of Molecular Ecology, Max Planck Institute for Chemical Ecology, Hans-Knöll-Str. 8, 07749 Jena, Germany; 3 Department of Entomology, Max Planck Institute for Chemical Ecology, Hans-Knöll-Str. 8, 07749 Jena, Germany; 4 These authors contributed equally to this work.

## Abstract

The olfactory response of the vinegar fly *Drosophila melanogaster* to food odor is modulated by starvation. Here we show that this modulation is not restricted to food odors and their detecting sensory neurons but rather increases the behavioral response to odors as different as food odors, repellents and pheromones. The increased behavioral responsiveness is paralleled by an increased physiological sensitivity of sensory neurons regardless whether they express olfactory or ionotropic receptors and regardless whether they are housed in basiconic, coeloconic, or trichoid sensilla. Silencing several genes that become up-regulated under starvation confirmed the involvement of the short neuropeptide f receptor in the starvation effect. In addition it revealed that the CCHamide-1 receptor is another important factor governing starvation-induced olfactory modifications.

Modulation and plasticity are key features of all organisms for adapting to e.g. a changing environment, stress, and food availability. Examples are blood-feeding insects, which after a blood meal switch their olfactory preference from host odors to odors specific for oviposition sites^1^,^2^,^3^. Accompanying this behavioral switch, receptors sensitive to lactic acid, a host-attractant substance, become desensitized^1^, while receptors sensitive to odors specific for oviposition sites become more sensitive^1^. Similarly in the African cotton leaf worm *Spodoptera littoralis*, the sensitivity of sensory neurons detecting feeding-related flower odors is down regulated upon mating, while the sensitivity of neurons detecting oviposition-related green leaf odors is up regulated^4^. We used the olfactory circuit of a well-established model, *Drosophila*^5^,^6^,^7^,^8^, to investigate whether feeding status modulates the flies' physiological and behavioral responses to odors. The main peripheral part of the olfactory circuit of *Drosophila* is housed in sensilla on the third antennal segment, where volatiles are detected via ca. 1200 olfactory sensory neurons (OSN). The OSNs are equipped with one of 62 olfactory receptor types and are found in stereotyped combinations of one-to-four OSNs in three morphological types of sensilla^8^. Root and coworkers showed that starvation increases the behavioral response and physiological sensitivity of *Drosophila* to the attractive food blend of apple cider and that this starvation-induced modulation is mainly governed by increased expression of the sNPF receptor in OSNs expressing the olfactory receptor OR42b^9^. Here, we illustrate that the starvation effect is neither restricted to these OSNs nor to food odors. It rather occurs in different OSN types, which express different olfactory receptors or even an ionotropic receptor. We furthermore confirm the role of the short neuropeptide f receptor (sNPF) and additionally establish the role of the CCHamide-1 receptor (CCHamide1r) in governing the starvation-induced modulation of fly olfactory responses.

Starvation affects the behavior towards stimuli as distinct as food odorants, repellents and a pheromone. Hence, starved flies found to be tuned not only to locate potential food sources from long distance, but also to evaluate the food quality and the presence of conspecifics efficiently.

## Results

### Starvation-induced changes in behavior

**Figure 1:**
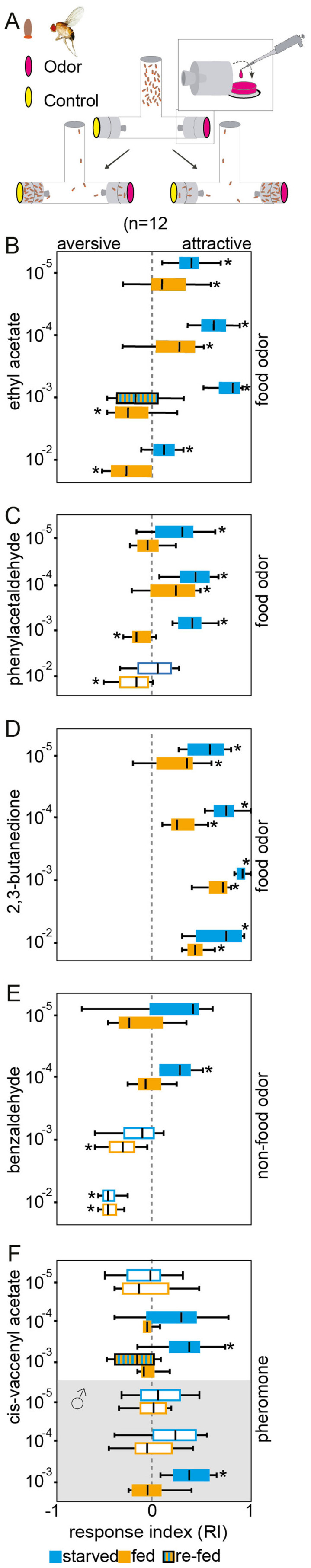
Behavioral responses of starved and fed flies. A. T-maze paradigm. 30 female flies were introduced into the center arm. Traps at the end of the T-arms were filled with an odor (red) or with solvent only (yellow). After 40 min flies in each trap and flies that did not enter any trap were counted and the response index calculated as RI = (#flies in odor trap – #flies in solvent trap)/#total flies. B–F. Fly choice in the T-maze tested with different concentrations of 5 odors. Grey shaded area in F, experiment performed with male flies. Box plots give the median (black bold line), 2^nd^ and 3^rd^ quartiles (box), and minima and maxima (whiskers) of twelve replicates. Filled boxes represent experiments with significant differences between starved and fed flies (Mann-Whitney-U test, p < 0.05); asterisks depict experiments where the RI values differed significantly from 0 (i.e. the odor was attractive or repellent).

We tested female flies in a T-maze paradigm (Fig. 1A) with the food odorants ethyl acetate and phenyl acetaldehyde. The odorants were attractive to fed flies only at medium concentrations and became repellent at high concentrations. However, starved flies were more strongly attracted than fed flies to all concentrations and were not repelled by high concentrations (Fig. 1B + C). We found an increased behavioral response to ethyl acetate already after few hours of starvation, but the effect increased with prolonged starvation time (Fig. S1). Contrary to these two odorants, 2,3-butanedione, another food-related odor, attracted fed flies in low as well as in high concentrations. However, again starved flies were significantly more attracted to all concentrations tested (Fig. 1D).

In order to test whether this increased behavioral responsiveness of starved flies was restricted to food odors only, we repeated the experiments with benzaldehyde, a well known repellent for *Drosophila*^10^,^11^. The fed flies did not respond to this odorant at low concentrations, while the starved flies became attracted to it. However, at high concentrations benzaldehyde repelled both starved and fed flies (Fig. 1E). Finally we tested cis-vaccenyl acetate (*c*VA), a pheromone that is involved in aggregation^12^, aggression^13^, and mating behavior^14^. While *c*VA was neutral to fed flies at all concentrations, it was attractive to starved flies at high concentrations (Fig. 1F). Since *c*VA regulates aggression and mating in male *Drosophila*, we additionally tested males. Like females, males became attracted to *c*VA only when they were starved before (Fig. 1F). To sum up, regardless which odors we tested we found an increased responsiveness in starved flies. Future studies will reveal whether this increased responsiveness of starved flies can also be observed towards odors that are sensed by sensory neurons expressing only one specific receptor type and are further processed via labeled lines like described for CO_2_^15^,^16^ and geosmin^17^.

### Starvation-induced changes in peripheral olfactory sensitivity

**Figure 2:**
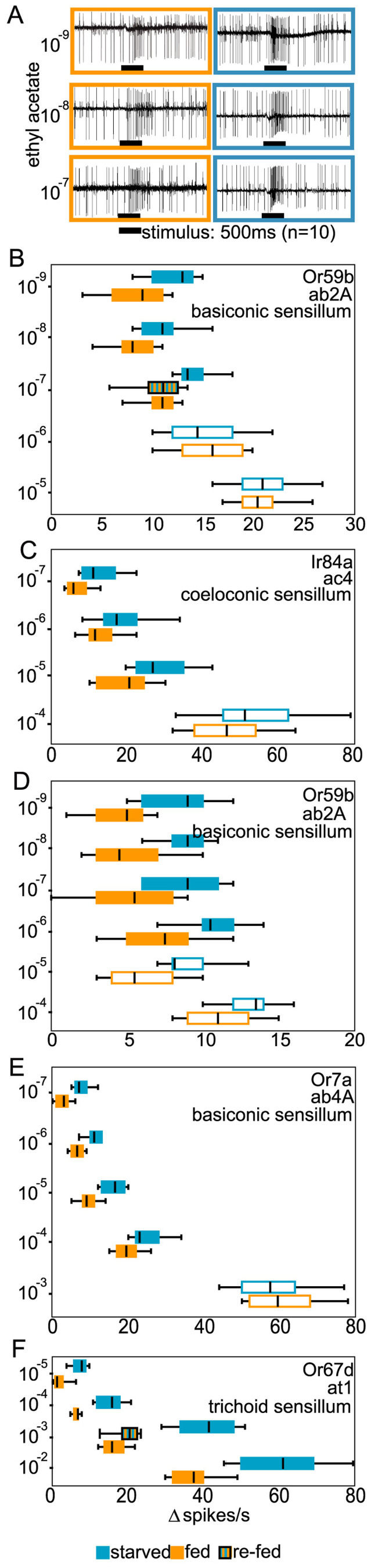
Physiological responses of starved and fed flies. A. Example spike traces from an ab2 sensillum stimulated with different concentrations of ethyl acetate. B–F. OSN changes in spike frequencies. (spike frequency during 1 s after stimulus onset) minus (spontaneous spike frequency during 1 s before stimulus onset) when stimulated with different concentrations of the same odors as in B–F. Single sensillum recordings from starved and fed flies. Box plots summarize the results recorded from each 10 sensilla. OSN and sensillum types are given in the top right corner of each plot. Orange: fed flies; blue: starved flies; striped: re-fed flies. In all cases re-fed flies were significantly different from starved flies but not from fed flies.

**Figure 3:**
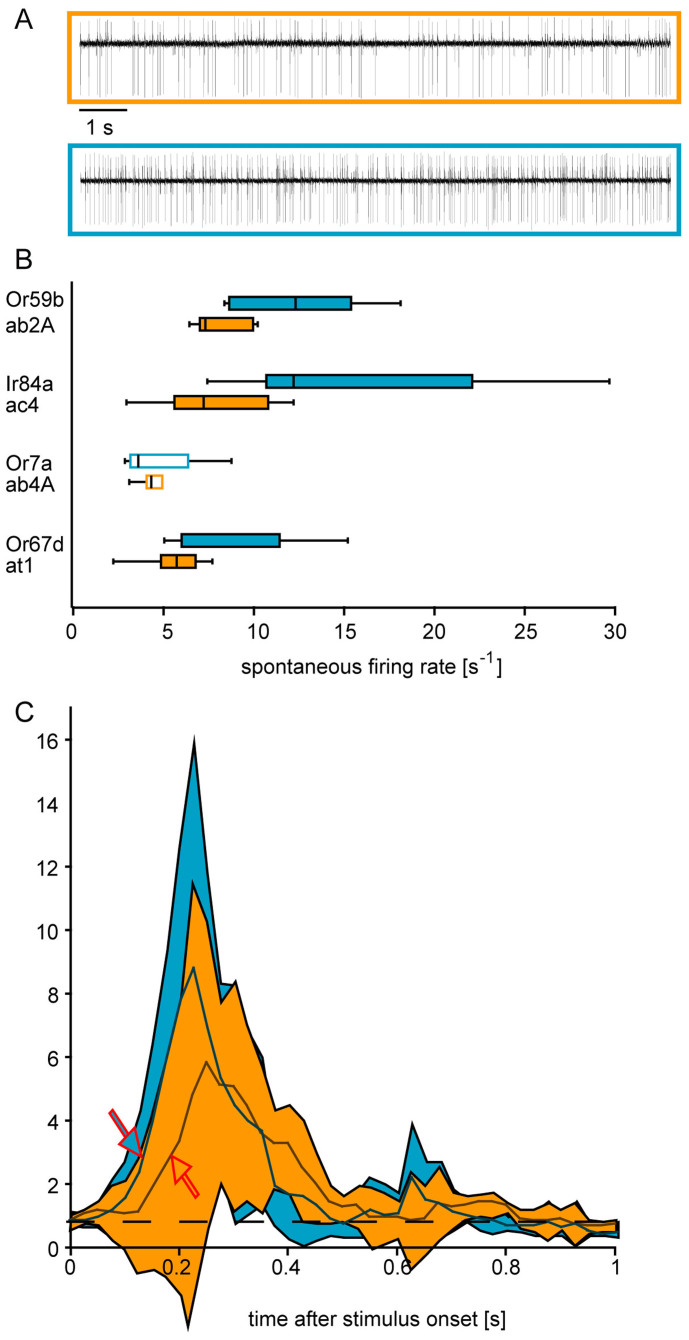
Spontaneous firing rate and response latencies of OSNs in starved and fed flies. A. Examples of spontaneous spike traces from OSNs housed in sensillum ab2. B. Starvation-induced changes in spike frequencies of different OSNs. C. Mean normalized response profile of OSNs in 10 ab2A sensilla to 500 ms pulses of ethyl acetate (diluted 10^−7^ in paraffin oil). Average firing rate before the stimulus was set as 1. Firing rates after the stimulus were normalized accordingly. Arrows indicate where the response becomes significantly higher than before the stimulus (Wilcoxon signed ranks test, N = 10, p < 0.05, Lines, average response; shaded areas, standard deviation). Orange: fed flies; blue: starved flies.

We next asked whether the increased behavioral responsiveness observed in starved flies comes along with a change in physiological sensitivity. We performed single sensillum recordings (Fig. 2A) from OSNs expressing the main target receptors of the same set of odors that was used in the behavioral experiments. All odors evoked stronger physiological responses in starved flies with the effects being most pronounced at low odor concentrations (Fig. 2B–F). In addition to increased spike rates after stimulation, we found increased spontaneous firing rates (Fig. 3A and B) and reduced response latencies in starved flies (Fig. 3C and Fig. S2). Again, the starvation effect was found not restricted to the detection of food odors (ethyl acetate and 2,3-butanedione both mainly targeting Or59b in the basiconic sensilla ab2, and phenyl acetaldehyde mainly targeting Ir84a in the coeloconic sensilla ac4, Fig. 2B–D). It also occurred in OSNs detecting non-food odors like benzaldehyde (Or7a in the basiconic sensilla ab4, Fig. 2E) and the pheromone *c*VA (Or67d in the trichoid sensilla at1, Fig. 2F). Therefore starvation affected all sensillum types and both receptor types – olfactory receptors (OR) as well as ionotropic receptors (IR). It should be pointed out here, that changes in the peripheral sensitivity might not necessarily be sufficient to explain the observed behavioral changes. It might well be, but was not the subject of this study, that also higher-order neurons involved in further processing the olfactory information and – hence – in governing the olfactory response might be affected as well.

Interestingly, when starved flies were exposed to sucrose, the starvation effect was abolished and one hour after feeding, both the behavior of the flies (Fig. 1B and F) and their sensitivity (Fig. 2B and F) resembled that of fed flies.

### Genes involved in the starvation effect

To investigate the molecular basis of the starvation process, we compared gene expression at the level of the antenna and the brain for fed and starved flies. After 28 hours of starvation, the expression of 209 genes in the antennae and of 999 genes in the brain was up regulated, while the expression of 47 genes in the antennae and 372 genes in the brain was down regulated (Table S1, FDR = 0.05). We found e.g. the expression of the short neuropeptide F 2.3times and that of the neuropeptide allatostatine 3.6times upregulated within the antenna, while the expression of CCHamide was 2.5times upregulated in the brain. We next focused on some of the genes that are known to be involved in the synthesis of these neuropeptides (allatostatine) or of the corresponding neuropeptide receptors (sNPFR1, CCHamide1r and AlstR).

**Figure 4:**
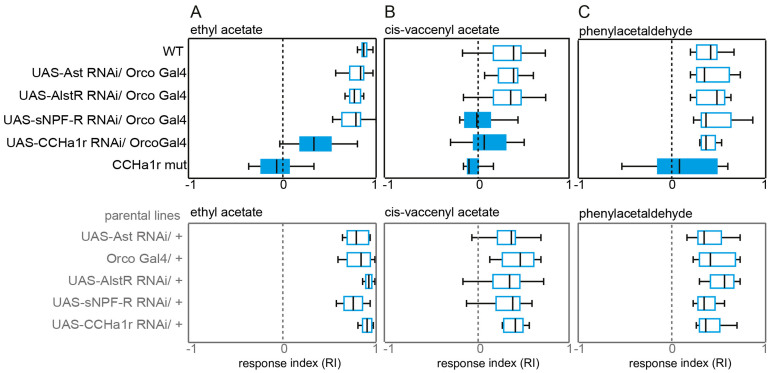
Impact of neuropeptides and their corresponding receptors on the starvation-induced modulation. A–C. Upper panels, behavioral responses of starved flies with silenced genes for neuropeptides or corresponding receptors to ethyl acetate (A), cis-vaccenyl acetate (B), and phenylacetaldehyde (C). Lower panels, olfactory behavioral responses of parental lines. For T-maze paradigm and explanation of box plot representation see Fig. 1. Filled boxes represent experiments with significant differences between mutant and wild type flies (one way ANOVA followed by tukey test, p < 0.05).

We silenced these genes at the level of the OR-expressing OSNs by using UAS-*RNAi* and Orco-Gal4 driver lines. We then tested if the flies still exhibited a starvation-induced increased behavioral responsiveness despite the silenced target genes (Fig. 4A–C). When we tested the behavior of starved flies to ethyl acetate (mainly detected by OR59b) silencing allatostatine, its corresponding receptor, or the sNPF receptor did not affect the starvation effect. Only silencing the CCHamide1 receptor resulted in an abolished starvation effect, suggesting a major role of CCHamide1 or its corresponding receptor in starvation-induced modulation in OR59b-expressing OSNs (Fig. 2A). Although sNPFR1 has been reported to govern the starvation effect (Root et al. 2011), sNPF seems not to be expressed in OR59b^18^. Therefore, we did not expect that the silencing of the sNPF receptor would affect the starvation-induced increased response to an odorant sensed by this neuron. The picture changed when we tested starved flies with cis-vaccenyl acetate (Fig. 2B). This compound is mainly detected by OSNs carrying OR67d, the neurons that have been shown to express sNPF^18^. As expected, silencing the sNPF receptor abolished the starvation-induced increased response to cVA, which confirms the involvement of this receptor in the starvation-induced modulation as shown before^9^. Again silencing the CCHamide1 receptor reduced the starvation effect, emphasizing the important role of this receptor in governing starvation-induced modulation. As expected, silencing the sNPF or the CCHamide1 receptor in OR-expressing OSNs did not affect the flies' responses to phenylacetaldehyde (Fig. 4C), which is mainly detected by OSNs expressing ionotropic receptors^19^. Only when we used CCHamid1r-mutant flies (i.e. flies that lacked the receptor not only in the olfactory but also in the ionotropic receptors), the starvation effect towards the IR-detected odorant phenylacetaldehyde was abolished (Fig. 4C).

## Discussion

*Drosophila melanogaster* uses olfaction to find and evaluate food sources^20^. Starved flies have been shown to be more attracted and more sensitive to the smell of cider vinegar^9^. Root and coworkers showed that the starvation-induced up-regulated neuropeptide receptor sNPFR1 in some of the OSNs activated by vinegar odor is responsible for the increased physiological and behavioral response to this odor.

Our finding that starved flies respond stronger to all tested odorants (Fig. 1) suggests that this starvation-induced modulation of olfaction is not restricted to food odors but is found also in the perception of odors that are significant in other contexts, like the repellent benzaldehyde or the pheromone cis-vaccenyl aldehyde. Following the finding of Root and coworkers that the expression of sNPFR1 is necessary and sufficient to explain the starvation-induced modulation, we expected that the behavioral response towards odorants that are mainly detected by neurons that do not express sNPF should not exhibit any starvation-induced modulation.

However, the behavioral response was up-regulated upon starvation not only for key ligands of the sNPF expressing neuron (cis-vaccenyl aldehyde targeting OR67d), but also for ligands of neurons which do not express sNPF (Carlsson et al. 2010; ethyl acetate and 2,3 butanedione, both targeting OR59b; benzaldehyde targeting OR7a; phenylacetaldehyde targeting IR84a). Hence, starved flies respond stronger to all odorants, regardless whether they are mainly detected by sNPFR1-expressing neurons or not. Therefore, beside the sNPF receptor, additional factors could be involved in the starvation-induced modulation. However most odorants are sensed by neurons expressing different receptor types (e.g. ethyl acetate is one of the main ligands of OR59b but is also detected by OSNs expressing 22a, 43b, and 47a^6^. Therefore, it cannot be ruled out that one of the neuron types activated by ethyl acetate expresses the sNPF receptor and hence, governs the up-regulated behavioral response.

By performing single sensillum recordings with the same set of odorants, that was used for the behavioral experiments, we found that starved flies exhibit an increased physiological response to low concentrated odorants (Fig. 2). Root and coworkers showed that in *Drosophila* the olfactory-driven activity of some OSNs and the corresponding projection neurons increases upon starvation^9^, while Farhadian and coworkers did not find any starvation-induced sensitization when recording from OSN that expressed OR47a^21^. Our data are in accordance with Root and coworkers but are contradictory to those of Farhadian and coworkers. However, one should bear in mind that we found the strongest difference in OSN responses of fed and starved flies when we tested odorants at low concentrations that were by 2–3 orders of magnitude lower than the concentrations used by Farhadian^21^. Therefore, we cannot say whether the conflicting results are caused by the different OSN types both studies recorded from or are due to the starvation effect becoming significant only at very low stimulus concentrations.

Biogenic amines have been shown to regulate a wide range of behavior including foraging^22^, circadian rhythms^23^ and sexual interactions^24^. In *Drosophila* it has been shown that sNPFR is involved in up-regulation of olfactory sensitivity due to starvation^9^. However, only about 25% of the *Drosophila* OSNs seem to express the sNPF receptor^25^. By silencing several genes that are known to be involved in the synthesis of neuropeptides or neuropeptide receptors, we could confirm the involvement of the sNPF receptor (Fig. 4). However, our observations indicate that starvation-induced modulation is not restricted to the OSNs expressing this gene. In addition the CCHamide1 receptor is involved in this modulation also (Figure 4). This explains, why OSNs – regardless whether they express ionotropic or olfactory receptors and regardless in which sensillum type they are housed – become sensitized upon starvation. This rather globally working starvation effect might increase the efficiency of starved flies to localize and evaluate food sources.

## Methods

### Flies

We used flies of the following lines: CantonS, UAS-s-NPFR *RNAi*, UAS-AST *RNAi*, UAS-AST-R *RNAi*, UAS-CCHa1r *RNAi*, Or83b GAL4, Mi{ET1}CCHa1^MB11962^ (Bloomington).

### Fly rearing and maintenance

Flies were maintained at 25°C, 70% relative humidity under 12L:12D in standard food vials (25 mm × 95 mm) containing standard cornmeal. Newly hatched flies (0–12 hrs old) were transferred to an odor-reduced medium^26^ and from now on were kept at 20°C. The flies were transferred to fresh medium every day in order to reduce pre-experimental olfactory experience. At the age of 4 days, female flies were collected and starved for 28 hours in a glass vial containing a moist bed of tissue paper (from now on referred to as “starved flies”). A second group of flies was kept under the same conditions but with access to 3% sucrose (“fed flies”). A third group of flies was starved for 27 hours and had access to 3% sucrose during the subsequent hour (“re-fed flies”).

### T-maze paradigm

Experiments were performed with a T-maze in which flies could enter either a trap that contained an odor or a control trap filled with solvent (Fig. 1A, for a detailed description see^17^). Thirty female flies (unless stated otherwise) were introduced to the maze and their position was scored after 40 minutes. We calculated the olfactory response index (RI) as described in legend of Fig. 1. The index could range from −1 (complete avoidance) to 1 (complete attraction). A value of 0 characterizes no response, i.e. the odor is not detected or is neutral. Each experiment was repeated 12 times and the RIs of starved and fed flies were compared with the Mann-Whitney-U test and tested against 0 (no response) by the Wilcoxon-rank-sum test.

### Single sensillum recordings

We performed single sensillum recordings from basiconic, coeloconic, and trichoid sensilla as described by^7^. Odor stimulation was performed as described by^18^ with slight modifications. A glass tube ended 15 mm from the antenna and supplied humidified air (9 ml/min). Odors were diluted in paraffin oil. In order to avoid cross-contamination, we used disposable pasture pipettes to add the odor stimuli (head space of 10 μl of the diluted odor on filter paper, duration 500 ms, controlled via Syntech CS55). All chemicals used in this study were delivered by Sigma-Aldrich (Stenheim, Germany) with the highest purity available (90–99%). Spike frequencies were analyzed for one second before and after the stimulation onset with the software Auto Spike v 3.2 (Syntech, Hilversum, The Netherlands). The response was calculated as spike frequency after stimulation – spike frequency before stimulation. The identification of different OSNs in a single sensillum was performed by spike sorting, i.e. based on differences in the spike amplitudes.

### RNA extraction and microarray analysis

Both for starved and fed flies RNA extraction was done four times each with 100 Antennae and 50 brains from 50 flies using the Qiagen RNA extraction kit. RNA concentration was measured photometrically with a NanoDrop ND-1000 and RNA quality and integrity was controlled with the Agilent 2100 Bioanalyzer. RNA was labeled with cyanine 3-CTP dye using the Low RNA Input Linear Amplification kit according to manufacturer instructions (Agilent Technologies). Labelled amplified cRNA samples were analyzed on a Nanodrop spectrophotometer using the microarray function and used for microarray hybridization at 65°C for 17 hours. Slides were washed, treated in stabilization and drying solution, scanned with the Agilent Microarray Scanner, and data was extracted with Agilent Feature Extraction software version 9.1. The resulting gene expression profiles were analyzed using GeneSpring GX software (Silicon Genetics, Redwood City, CA). Raw intensities were normalized using the 75th percentile value and log2 and baseline transformed prior statistical analysis. We performed the implemented t-test for comparing two samples at a time and corrected the p-value for multiple testing. The microarray data with each probe name was deposited in the NCBI GEO database (accession number GSE48077).

## Author Contributions

Behavioral experiments were designed and analysed by A.F., B.S.H. and M.K. Behavioral experiments were conducted by A.F. Microarray analysis was conducted and analysed by A.F., J.G., E.G.W. and H.V. Manuscript was written by A.F., B.S.H. and M.K. All authors reviewed the manuscript.

## Supplementary Material

Supplementary Information Supplementary Information
