# Supplementary material for: The CCHamide 1 receptor modulates sensory perception and olfactory behavior in starved Drosophila
Source: Sci Rep. 2013 Sep 26;3:2765. doi: 10.1038/srep02765 (PMC3783891; doi:10.1038/srep02765)
Supplement: Supplementary Information [file srep02765-s1.pdf]

The CCHamide 1 receptor modulates sensory perception and olfactory behavior in starved *Drosophila*

Abu Farhan, Jyotasana Gulati, Ewald Große-Wilde, Heiko Vogel, Bill S. Hansson, and Markus Knaden

Supplementary Information

Figure S1

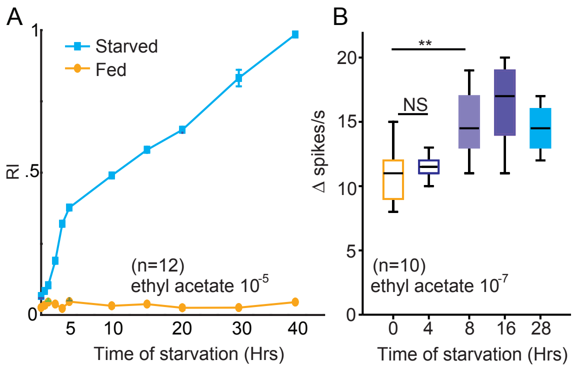

Figure S1. Starvation effect increases with prolonged starvation. A. Behavioral responses of flies to ethyl acetate that were starved between 1 and 40 hours. For explanation of T-maze paradigm and box-plot representation see Fig. 1. B. Odor-driven activation of an OSN expressing OR59b of flies that were starved between 0 and 28 hours. For the explanation of box plots see Fig. 3.

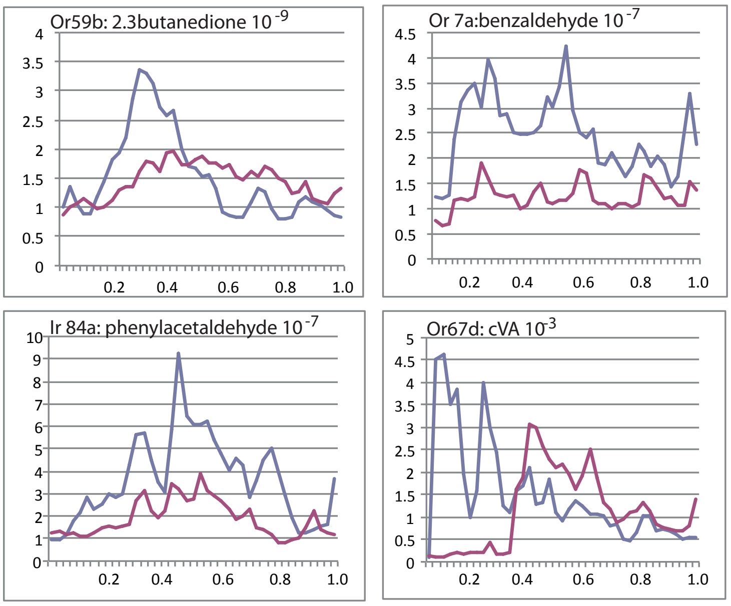

Figure S2. Mean normalized response profiles of OSNs in each 10 sensilla to 500ms pulses of different odorants. Average firing rate before the stimulus was set as 1. Firing rates after the stimulus were normalized accordingly.
